# Development of SNP assays for hessian fly response genes, *Hfr-1* and *Hfr-2*, for marker-assisted selection in wheat breeding

**DOI:** 10.1186/s12863-018-0659-y

**Published:** 2018-07-31

**Authors:** Mui-Keng Tan, Mustapha El-Bouhssini, Ossie Wildman, Wuletaw Tadesse, Grant Chambers, Shuming Luo, Livinus Emebiri

**Affiliations:** 1 NSW Department of Primary Industries, Elizabeth Macarthur Agricultural Institute, Woodbridge Road, Menangle, NSW 2568 Australia; 2 The International Center for Agricultural Research in the Dry Areas (ICARDA), Rabat Instituts, P.O. Box 6299, Rabat, Morocco; 3 NSW Department of Primary Industries, Wagga Wagga Agricultural Research Institute, Pine Gully Road, Wagga Wagga, NSW 2650 Australia; 4 Graham Centre for Agricultural Innovation (NSW Department of Primary Industries and Charles Sturt University), Wagga Wagga, NSW 2650 Australia

**Keywords:** Wheat, SNP markers, Hessian fly response genes, Pest resistance, Dirigent protein, Effectors, Lectin domain

## Abstract

**Background:**

The Hessian fly response genes, *Hfr-1* and *Hfr-2,* have been reported to be significantly induced in a Hessian fly attack. Nothing is known about the allelic variants of these two genes in susceptible (S) and resistant (R) wheat cultivars.

**Results:**

Basic local alignment search tool (BLAST) analysis of Hessian fly response genes have identified three alleles of Hessian fly response gene 1 (*Hfr-1*) on chromosome 4AL and 7DS, and 10 alleles of Hessian fly response gene 2 (*Hfr-2*) on chromosome 2BS, 2DL, 4BS, 4BL, 5AL and 5BL. Resequencing exons of *Hfr-1* and *Hfr-2* have identified a single nucleotide polymorphism (SNP) in the lectin domain of each gene that segregates some R sources from S cultivars. Two SNP assays have been developed. The SNP883_*Hfr-1* assay characterizes a ‘G/A’ SNP in *Hfr-1*, which differentiates 14 Hessian fly R cultivars from S ones. The SNP1294_*Hfr-2* assay differentiates 12 R cultivars from S ones. Each of the two SNPs identified in *Hfr-1* and *Hfr-2* is ‘G/A’ and resulted in an amino acid change from isoleucine to valine in the lectin domain of the proteins of the alleles in the R cultivars. In addition to the genotype profiles of *Hfr-1* and *Hfr-2,* generated for a set of 249 wheat cultivars which included a set of 39 R cultivars, this study has genotyped the Hessian fly response gene, *HfrDrd,* and the *H32* gene for the wheat germplasm. Resistant cultivars from different origins with one, two, three or four resistance (*R*) genes in various combinations/permutations have been identified.

**Conclusion:**

This study has identified allelic differences in two Hessian fly response genes, *Hfr-1* and *Hfr-2*, between S and R cultivars and developed one SNP assay for each of the genes. These two SNP assays for *Hfr-1* and *Hfr-2,* together with the published assays for *HfrDrd* and the *H32* gene, can be used for the selection and incorporation of one or more of these 4 *R* genes identified in the different R sources in wheat breeding programs.

**Electronic supplementary material:**

The online version of this article (10.1186/s12863-018-0659-y) contains supplementary material, which is available to authorized users.

## Background

The Hessian fly, [*Mayetiola destructor* (Say)] is one of the most destructive pests of wheat in North America, North Africa, southern Europe and northern Kazakhstan. Experiments in Oregon USA reported loss of yield by as much as 59% in S cultivars compared to R lines when as low as 15% of the plants were infested [1].

Hessian fly interacts with wheat in a way similar to many plant-pathogen interactions in a typical gene-for-gene relationship [2]. To date, at least 34 Hessian fly *R* genes, designated *H1* through *H34*, have been described in wheat and its relatives [2–9]. The occurrence of numerous Hessian fly *R* genes identified in the A, B and D genomes of wheat suggests a long co-evolutionary relationship between wild wheat species and the Hessian fly.

These Hessian fly *R* genes, *H1* through *H34*, had been identified through the genetic analysis of suitable crosses derived from R cultivars [7–9]. These *R* genes had been located following constructions of linkage groups in the mapping populations and quantitative trait locus (QTL) analysis. Multiple mapping populations have to be developed to locate the numerous *R* genes associated with various R genotypes. Further research work has to be performed to develop and identify user-friendly molecular markers linked to various mapped *R* genes [10] for implementation in high-throughput marker-assisted selection in breeding. This approach is extremely time-consuming, labor intensive and very costly.

Hessian fly larvae are believed to inject salivary proteins into wheat tissue [11], capable of being detected as avirulence factors in a classic gene-for gene interaction [12, 13] if the plant contains a corresponding *R* gene. Larval virulence can be restored if the elicitor from the saliva is altered in its molecular form to avoid detection, resulting in elimination of the defense trigger. Continuous large scale cultivation of a wheat cultivar with a specific *R* gene exerts strong pressure for the selection of virulent pest genotypes. *R* genes have been reported to be ineffective after 8–10 years of deployment [14]. Continuous evolution of virulent genotypes necessitates the identification of new *R* genes from diverse origins and sources.

This study analyzed two Hessian fly response genes, *Hfr-1* and *Hfr-2,* which have been reported to be specifically and significantly induced in a Hessian fly attack. The objective was to identify allelic variants in these genes that can be utilized for their selection in breeding.

The Hessian fly-response genes, *HfrDrd* and *Hfr-1*, were reported to be induced specifically in response to infestation by Hessian fly larvae [15–18]. *HfrDrd* is a dirigent-like protein gene, while the *Hfr-1* gene (GenBank: AF483596.1) has two domains; a N-terminal region that shares sequence similarity with disease resistance response dirigent-like protein gene and a C-terminal sugar binding jacalin-like lectin domain. A SNP in the conserved dirigent domain of the *HfrDrd* gene [19], which caused a serine to leucine change in the dirigent-like protein, has been found to be significantly associated with Hessian fly R cultivars. A SNP assay has been developed for the selection of *HfrDrd* gene [19].

Dirigent and dirigent-like proteins are optically active [20] and have been reported to play a significant role in membrane remodeling and cell-wall strengthening of the plant barrier at the attack site to restrict and/or prevent the larvae from feeding on the plant [18, 21–23] with a significant drastic impact on insect growth and development.

In addition to a dirigent-like domain, *Hfr-1* has a lectin-like domain and thus this gene has bi-functional biological roles [24]. Lectins belong to a complex group of proteins and possess structural diversity with different affinity for several carbohydrates [24]. They play a role in defence in pathogen recognition and as feeding deterrents against insects in plants [25, 26].

Another lectin containing protein coded by *Hfr-2* (AY587018.1) had been reported to be up-regulated in response to infestation by Hessian fly larvae [27]. The lectin domain of *Hfr-2* (AY587018.1) was postulated to act as a toxin to the gut of the insects and negatively impact the growth and development. It was also proposed that as the sequence is similar to channel forming toxins and cytolytic toxins, the protein may be used by the pest to their advantage to form pores at the attack site to provide nutrients to the pest [27]. Experimental evidence had indeed shown that Hessian fly larvae do not inflict mechanical damage to the root tissue of wheat hosts, but establish a channel for feeding on the liquid nutrients from the host [28]. Thus a mutant form resulted from one or more nucleotide changes in the lectin domain can change the protein’s response to the salivary effectors, which will either act as an insect toxin and/or hinder the formation of a nutrient channel for the larvae and thus acts as a *R* gene.

This study has designed experiments to explore for informative SNPs located in conserved protein domains of *Hfr-1* and *Hfr-2* genes for their use in gene selection during breeding. The availability of the draft whole genome sequence data for wheat (http://www.wheatgenome.org) has enabled the exploration of the Hessian fly response genes for alleles in the entire wheat genome and the design of experiments to find informative SNPs that differentiate R sources from S ones. A single base-pair change in a target gene has been reported to change host resistance to chemical elicitors such as herbicides [29, 30] or biological elicitors from pathogens [31–33]. In these instances, a single nucleotide mutation in the conserved domain of the target gene that leads to an amino acid substitution, change the molecular form of the protein, resulting in changes in sensitivity to the elicitor molecules.

This study reports on the development of two SNP assays, one each for the selection of the Hessian fly response genes, *Hfr-1* and *Hfr-2*. These assays have been genotyped in combination with two SNP assays (synopGBS901 and IWB65911) linked to the resistance gene, *H32* [34] and a SNP assay for the Hessian fly-response gene, *HfrDrd* [19] on a set of S and R wheat germplasm to generate genotype profiles for various R cultivars from diverse origins. The genotype profiles of these Hessian fly *R* genes in R cultivars will be valuable for the selection of one or more of these genes for incorporation in wheat breeding for Hessian fly resistance.

## Results

### BLAST analysis of *Hfr-1*

**Fig. 1:**
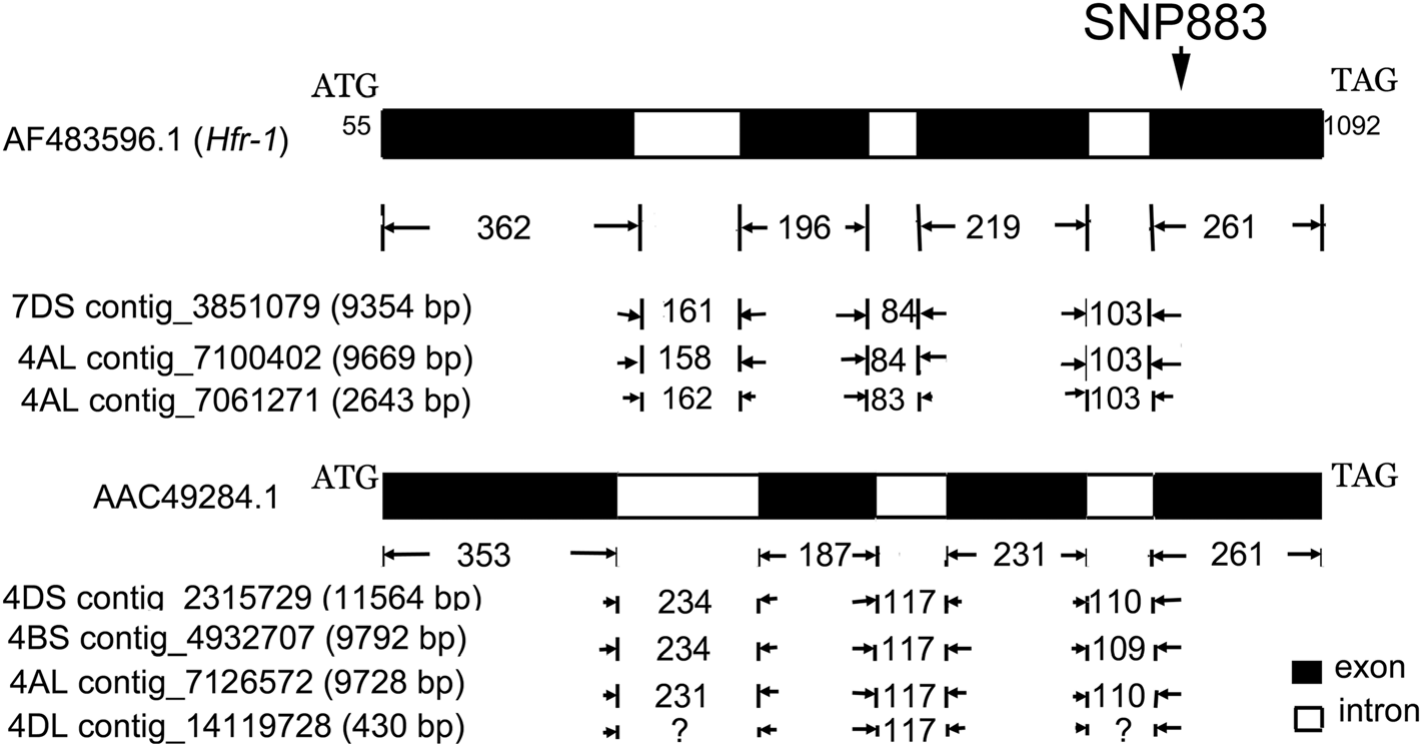
The *Hfr-1* (AF483596.1) gene comprises four exons and three introns. The numbers at start and end of the structure indicate the nt positions of the coding sequence in the *Hfr-1* mRNA sequence (AF483596.1). The 5′ and 3′ untranslated regions (UTR) are not shown. BLAST analysis with the wheat genome repository (https://wheat-urgi.versailles.inra.fr/Seq-Repository/) retrieved the complete genomic sequences of three alleles; one on chromosome 7DS and two on 4AL. The coding sequences on these 3 contigs are: 7DS contig 3,851,079 (9354 bp, Expect = 0) - (2067..2428, 2590..2785, 2870..3088, 3192..3452), 4AL contig 7,100,402 (9669 bp) - (6726..7087, 7246..7441, 7526..7743, 7847..8107) and 4AL contig 7,061,271 (2643 bp)- (1056..1404, 1567..1762, 1845..2063, 2167..2427). Allelic variations were observed in the sizes of intron I and 2, and the SNPs identified in the exons of the three alleles (Additional file 1: Table S1). Sequence alignment enabled the design of four primer pairs (Table 1) to sequence the exons of *Hfr-1* for wheat cultivars in this study. Analysis (see ‘Results’ and ‘Discussion’) facilitated the development of a SNP assay at nt 883 (SNP883_*Hfr-1* assay) for the selection of *Hfr-1* from resistant sources with the *R* allele. The closely related protein, AAC49284.1 also has four exons and three introns. BLAST analysis retrieved complete genomic sequences of three alleles on chromosomes 4DS, 4BS, 4AL and a partial sequence on chromosome 4DL. The coding sequences on these contigs are: 4DS contig 2,315,729 (11,564 bp, Expect = 3e^− 79^)- complement (4882..5142, 5253..5483, 5601..5787, 6022..6374); 4BS contig 4,932,707 (9792 bp) - complement (2403..2663, 2773..3003, 3121..3307, 3542..3894); 4AL contig 7,126,572 (9728 bp)- complement (1441..1701, 1812..2042, 2160..2346, 2578..2891) and a 4DL contig 14,119,728 (430 bp) – complement (< 1..91, 209..395>). Like the *Hfr-1* protein, this closely related protein has a dirigent domain and a jacalin-like lectin domain.

The protein of *Hfr-1* (AAM46813.1) has 345 amino acids with a dirigent domain (42..178) and a jacalin domain (206..340). BLAST analysis using the messenger RNA (mRNA) sequence of *Hfr-1* (AF483596.1) against the wheat genome repository (https://wheat-urgi.versailles.inra.fr/Seq-Repository/) retrieved the complete gene sequence on a chromosome 7DS contig 3,851,079 (9354 bp, Expect = 0), and two 4AL contigs, 7,100,402 (9669 bp) and 7,061,271 (2643 bp). Sequence alignment of the mRNA and the genomic contig sequences revealed the gene has 4 exons of 362, 196, 219 and 261 bp (Fig. 1). The three alleles on 7DS and 4AL differ in the sizes of the introns between exon I and exon II, and between exon II and exon III (Fig. 1). The intron between exons III and IV is similar in size in the three alleles (Fig. 1).

The *Hfr-1* mRNA sequence (AF483596.1) has very high similarity (2e-168) to an unknown wheat protein, AAC49284.1 (Fig. 1). This closely related protein with 343 amino acids, similarly has a dirigent domain (39..172) and a jacalin-like lectin domain (204..338). This protein was reported to be induced by Benzothiadiazole, a chemical that induces systemic acquired resistance and activates disease resistance in wheat [35].

BLAST analysis of the mRNA of the unknown protein (AAC49284.1) against the wheat genome repository (https://wheat-urgi.versailles.inra.fr/Seq-Repository/) has retrieved complete gene sequence on three contigs (Fig. 1). The three contigs with their corresponding coding sequences for the unknown protein (AAC49284.1) are: 4DS IWGS contig 2,315,729 (11,564 bp)- complement (4882..5142, 5253..5483, 5601..5787, 6022..6374); 4BS contig 4,932,707 (9792 bp)- complement (2403..2663, 2773..3003, 3121..3307, 3542..3894) and a 4AL IWGS contig 7,126,572 (9728 bp) - complement (1441..1701, 1812..2042, 2160..2346, 2578..2891). A partial gene sequence was retrieved on a 4DL IWGS contig 14,119,728 (430 bp)- complement (< 1..91, 209..395>). Alignment of the genomic sequences with the mRNA of the unknown protein (AAC49284.1) has revealed this protein similarly has 4 exons (Fig. 1). All except exon IV were different in sizes to the exons of *Hfr-1*.

### BLAST analysis of *Hfr-2*

**Fig. 2:**
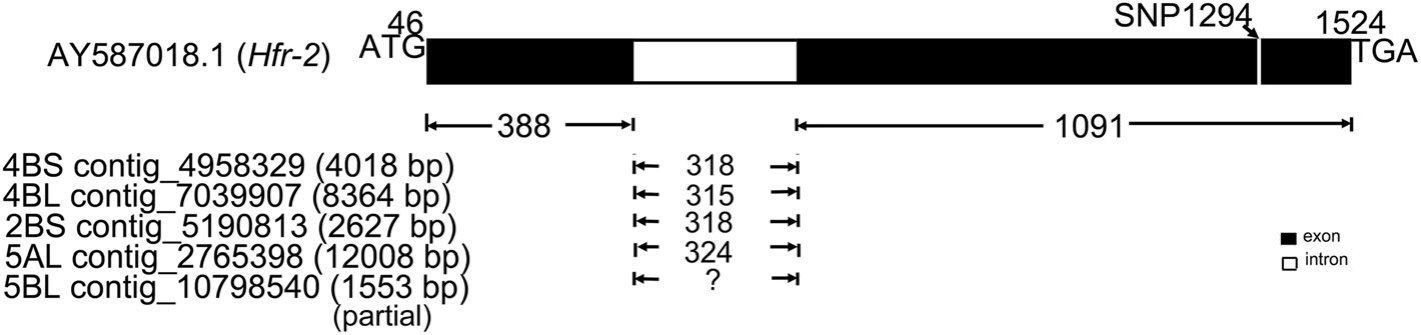
The *Hfr-2* (AY587018.1) gene comprises two exons and one intron. The numbers at start and end of the structure indicate the nt positions of the mRNA sequence in *Hfr-2* (AY587018.1). The 5′ and 3′ untranslated regions (UTR) are not shown. BLAST analysis with the wheat genome repository (https://wheat-urgi.versailles.inra.fr/Seq-Repository/) retrieved (Expect = 0) the complete genomic sequence of alleles on chromosomes 4BS, 4BL, 2BS and 5AL; and partial sequences of alleles on chromosomes 5BL, 4BS, 4BL and 2DL. The complete coding sequences on four contigs are: 4BS contig 4,958,329 (4018 bp) – complement (447..1537, 1856..2243); 4BL contig 7,039,907 (8364 bp) - (2916..3303, 3619..4709); 2BS contig 5,190,813 (2627 bp) - (714..1101, 1420..2510); 5AL contig 2,765,398 (12,008 bp) - (8239..8639, 8964..10053). The partial coding sequence on the 5BL contig 10,798,540 (1553 bp) - (< 27..1117>). Allelic variations were observed in the intron sizes and the SNPs identified in the exons of these 5 alleles (Additional file 1: Table S1). Sequence alignment enabled the design of three primer pairs (Table 1) to sequence the exons of *Hfr-2* for wheat cultivars in this study. Analysis (see ‘Results’ and ‘Discussion’) facilitated the development of a SNP assay at nt 1294 (SNP1294_*Hfr-2* assay) for the selection of *Hfr-2* from R sources. Contigs not illustrated are 4BS contig 4,958,328 (3162 bp)-complement (447..1537, 1858..2245); 4BS contig 4,888,577 (1419 bp)- (< 1..973), partial exon II; 2DL contig 9,762,580 (542 bp)- (< 26..542>), partial exon II; 4BL contig 7,014,725 (700 bp)- complement (< 1..700>), partial exon II; 4BL 7,009,722 (1814 bp)- complement (< 306..692>), partial exon I. Ten alleles of this gene have been identified

BLAST analysis using the mRNA sequence of *Hfr-2* (AY587018.1) against the wheat genome repository (https://wheat-urgi.versailles.inra.fr/Seq-Repository/) retrieved (Expect = 0) the complete gene sequence on five contigs on chromosomes 4BS, 4BL, 2BS and 5AL. The five contigs included two 4BS contigs, 4,958,329 (4018 bp) and 4,958,328 (3162 bp), a 4BL contig 7,039,907 (8364 bp), a 2BS contig 5,190,813 (2627 bp) and a 5AL contig 2,765,398 (12,008 bp, Fig. 2). A sequence comprising exon II and the partial intron was found on a 5BL contig 10,798,540 (1553 bp, Fig. 2). A partial sequence of exon I was retrieved on a 4BL contig 7,009,722 (1814 bp) and partial sequences of exon II was found on a 2DL contig 9,762,580 (542 bp), a 4BS contig 4,888,577 (1419 bp) and a 4BL contig 7,014,725 (700 bp).

Sequence alignment of the mRNA and the genomic contig sequences revealed the gene has two exons of 388 and 1091 bp (Fig. 2). The single intervening intron for the alleles varies in size, from 315 bp (4BL, 4BS), 318 bp (2BS, 4BS) to 324 bp (5AL). The alignment also revealed SNPs in the *Hfr-2* coding sequences on the three 4BS contigs and the three 4BL contigs (Additional file 1: Table S1); indicating the presence of three 4BS and three 4BL alleles. Thus a total of ten *Hfr-2* gene alleles have been found with three on 4BS, three on 4BL, one each on 2BS, 2DL, 5AL and 5BL (Additional file 1: Table S1). One and two primer pairs were designed to sequence exon I and exon II respectively (Table 1) in a set of S and R cultivars (see ‘Methods’).

**Table 1:** Sequences of primer pairs for the amplification of exon segments in *Hfr-1* and *Hfr-2* genes

Primer names	Primer sequence (5′ to 3′)	Amplicon size (bp)	Exon	Overall outcomes
*Hfr-1*				
Hfr-1_F1	CCTCAGTCTTTCACCTTGGAGACC	294	I	The sequences were poor.
Hfr-1_R1	CCCGAGGTGCAAACCTTGAGC			
Hfr-1_F2	GGTTTACAGGTTCCAGTTTCAAGG	205	II	7 SNPs occurred randomly across cultivars
Hfr-1_R2	CACCAGAGGTCTTAAGAAGGAGAG			
Hfr-1_F3	CCGAGTCCGACCAGCTTTCTC	237	III	7 SNPs were detected –some result in no amino acid change, some result in amino acid substitution but did not differentiate any R cultivar from S ones.
Hfr-1_R3	CTACCGTGTCCTTGTTATCCCCAC			
Hfr-1_F4	CCTTCAGAGATCGTTACGGAAGT	210	IV	8 SNPs were detected. The SNP at nt 883 (AF483596) was found to differentiate some R cultivars from S cultivars (Table 3).
Hfr-1_R4	CTCCACGTAYTTACCAGCGCAC			
*Hfr-2*				
Hfr-2_F1	GCAAGTACCTAGGTAGCGTGC	340	I	2 SNPs were observed to occur in random.
Hfr-2_R1	CGTACCTGATGGATAGATTATTGTCC			
Hfr-2_F2a	CTTCCGTCCYCAGCAGACAAC	678	II	29 SNPs were detected but were found across both S and R cultivars.
Hfr-2_R2a	GGTGTCATTGGTTGAAGTCATGCTGG			
Hfr-2_F2b	CTATGGCGTGGAGTTCAAGCTC	433	II	The SNP at nt 1294 (GenBank AY587018) was found to differentiate some R cultivars from S cultivars (Table 3). 16 other SNPs found were not informative.
Hfr-2_R2b	TCTTGTAGGTAACAACCTCTCCTG			

Refer to Additional file 1: Table S1 for details of the SNPs observed in the exon fragments of *Hfr-1* and *Hfr-2* of S and R wheat accessions sequenced

### Analysis of exon sequences of *Hfr-1* and *Hfr-2*

**Fig. 3:**
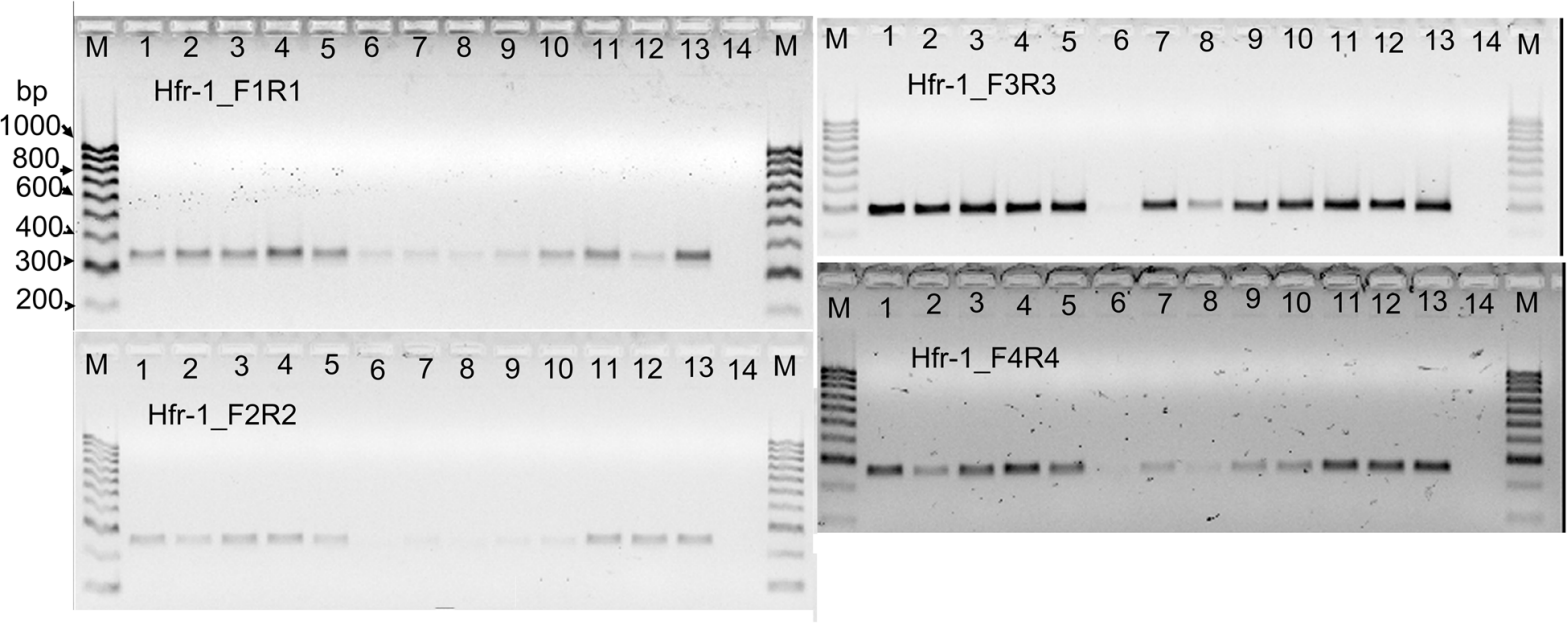
Amplicons of each of the four exons, I, II, III and IV, of *Hfr-1* using the appropriate primer pairs (Table 1) were all single fragments of expected sizes, 294, 205, 237 and 211 bp respectively. The samples sequenced were AGT-Young (1), Axe (2), Carnamah (3), Janz (4) Cham6 (5), Clark (6), Ella (7), AUS26929 (8), Monon (9), Newton (10), CIGM90.898 (11), CIGM90.906 (12) and CASW02GH00010S (13). Another 2 cultivars, Frame and Haala-35 were performed first before this second bigger lot of cultivars. Lane 14 is no template control and lane M is the marker of 100 bp ladder

**Fig. 4:**
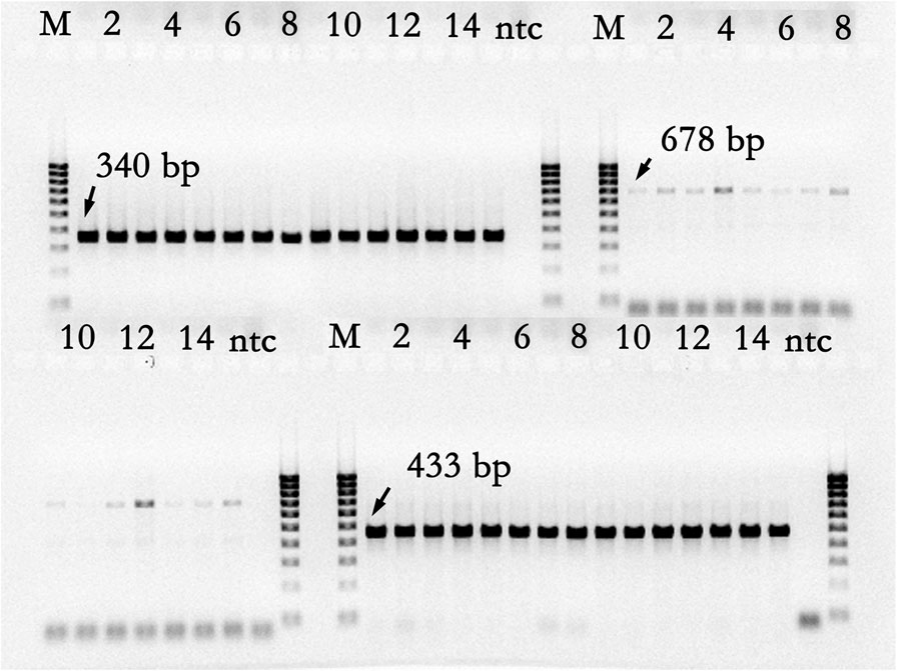
Amplicons of the two exons, I, IIa and IIb, of *Hfr-2* using the appropriate primer pairs (Table 1) were all single fragments of expected sizes; 340, 678 and 433 bp respectively. The samples sequenced were AGT-Young (1), Axe (2), Carnamah (3), Janz (4) Cham6 (5), Clark (6), Ella (7), AUS26929 (8), Monon (9), Newton (10), CIGM90.898 (11), CIGM90.906 (12), CASW02GH00010S (13), Frame (14) and Haala-35 (15). Lane ntc is no template control and lane M is the marker of 100 bp ladder

Fragments of expected sizes (Table 1) were obtained for each of the exon segments of the two Hessian fly response genes amplified from the wheat cultivars (Figs. 3, 4). The sequences obtained from sequencing each of the amplicons were aligned with the reference mRNA sequences of *Hfr-1* (AF483596.1) and *Hfr-2* (AY587018.1). Any SNP observed (Additional file 1: Table S1) for each of the sequenced amplicons of a wheat cultivar was referenced to the nucleotide (nt) positions of the reference mRNA sequences, *Hfr-1* (AF483596.1) and *Hfr-2* (AY587018.1).

**Fig. 5:**
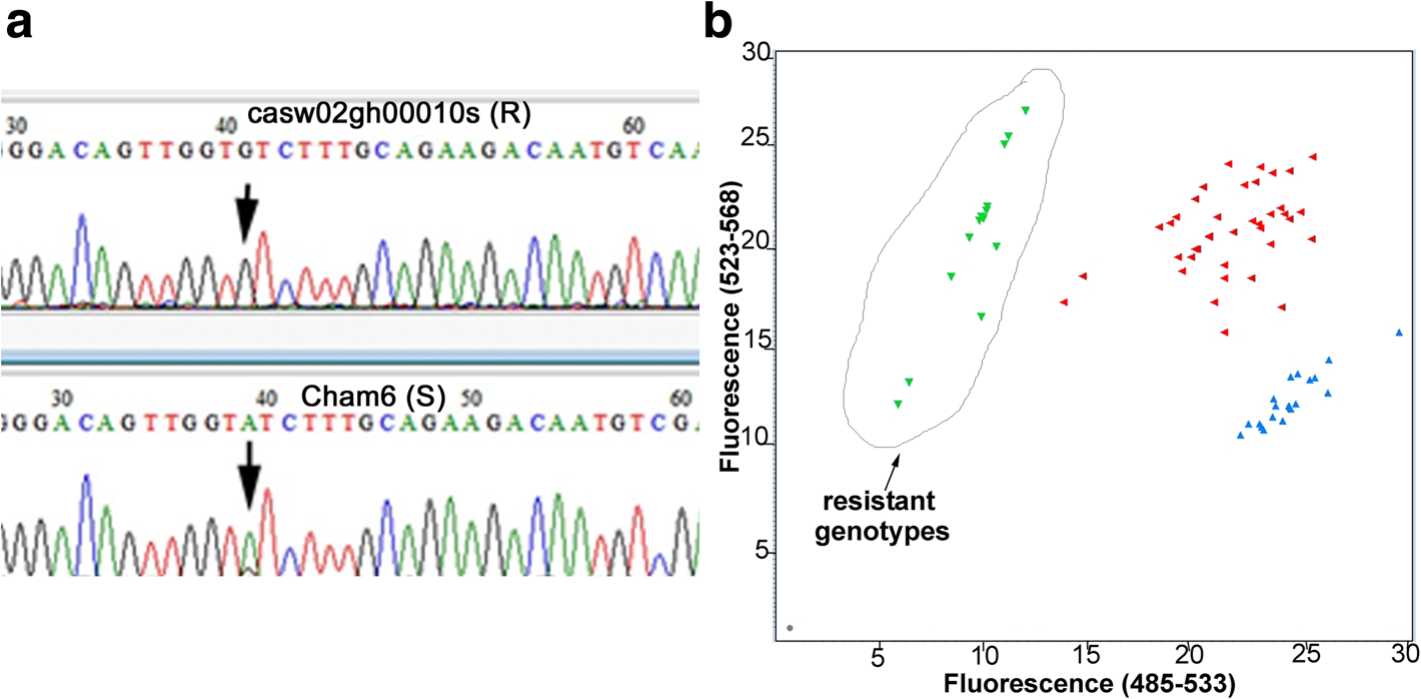
**a** Partial sequence profiles of amplicon from *Hfr-1* with primer pair Hfr-1_F4 and Hfr-1_R4 for 1 R cultivar, CASW02GH00010S, and 1 S cultivar Cham6. The indicated SNP, A/G is located at nt position 883 of *Hfr-1* mRNA, GenBank AF483596.1, which results in an amino acid substitution of isoleucine to valine. This SNP is located in the jacalin-like plant lectin domain of the gene. **b** The endpoint fluorescence in the genotyping of SNP 883 in exon IV of *Hfr-1* using the SNP assay, SNP883_*Hfr-1* (Table 2). This SNP segregates between the R cultivars with ‘G’ (VIC; green triangles on y-axis), and the S cultivars with either the ‘A’ (FAM; blue triangles on x-axis) or the heterozygotes (red triangles). This SNP results in an amino acid change from isoleucine to valine in R cultivars. The ‘G’ allele is present in 14 of the 39 R cultivars (Table 3). No S cultivar has the ‘G’ allele (Table 3)

Poor sequence data was obtained for exon I of *Hfr-1* for all cultivars (Table 1). A total of 22 SNPs were observed for the other three fragments of the *Hfr-1* gene sequenced (Additional file 1: Table S1), the numbers of SNPs being 7, 7 and 8 on exon II, exon III and exon IV respectively. Eleven of the SNPs do not result in an amino acid change and 10 of the SNPs occur randomly in both S and R cultivars (Additional file 1: Table S1). One SNP, ‘A/G’, nt 883 in AF483596.1, located in exon IV of *Hfr-1*, was observed to segregate with 2 R sources (CIGM90.898, CASW02GH00010S). These two sources have the nt, ‘G’ (Fig. 5a), whereas all the other cultivars sequenced have either the ‘A’ nucleotide (Fig. 5a) or the ‘A:G’ heterozygote genotype (Additional file 1: Table S1).

A Taqman assay, SNP883_*Hfr-1*, was designed for high throughput genotyping (Table 2). The set of Australian wheat cultivars, which are all S, gave either the ‘A:G’ or the ‘A:A’ genotype (Fig. 5b, Table 3, Additional file 2: Table S2). No ‘G:G’ genotype was observed for the S cultivars. In contrast, fourteen of the thirty nine R genotypes have the ‘G:G’ genotype (Fig. 5b, Table 3).

**Table 2:** SNP assays for *Hfr-1* and *Hfr-2*

Primers and Probes	Sequences (5′ to 3′)
SNP883_*Hfr-1* assay	
SNP883_Hfr-1_F	TTCAGAGATCGTTACGGAAGTTTCTG
SNP883_Hfr-1_R	GTGAGGGATGCTATAGCATTATATTCGA
SNP883_Hfr-1_probe1 (Vic)	ACAGTTGGTGTCTTTG
SNP883_Hfr-1_probe2 (Fam)	ACAGTTGGTATCTTTG
SNP1294_*Hfr-2* assay	
SNP1294_Hfr-2_F	GGTCATGGAGGCACCAC
SNP1294_Hfr-2_R	GCAGAAGGGAAGGTTGAGGTATC
SNP1294_Hfr-2_probe1 (FAM)	CGTTGTCTGAACGGATGA
SNP1294_Hfr-2_probe2 (CalFluor)	CGTTGTCTGAATGGATGA

**Table 3:** Genotype profiles of some R wheat cultivars for three Hessian fly response genes, *HfrDrd, Hfr-1* and *Hfr-2* and the *H32* gene

Wheat Accessions	# cultivars	Locality	Phenotype	^a^SNP143_*HfrDrd*	SNP883_*Hfr-1*	^b^SNP1294_*Hfr-2*	^c^synopGBS901 *H32*	^c^IWB65911 *H32*
Chrom location				7DS/7AS	7DS/4AL	2BS/2DL/4BS/4BL/5AL/5BL	3DL	3DL
^d^78 wheat cultivars	78	^d^various	^e^S	C:C	A:A	T:T	G:G	A:G
^d^131 wheat cultivars	131	^d^various	S	C:C	A:G	T:T	G:G	A:G
Sunguard	1	Aus	S	C:C	A:G	T:T	**C:G**	A:G
NS732/HER*2//Saada, Ouassou-18, Morsud-31, Miskeet-3, Miskeet-8, Miskeet-18, Haala-34, Doukkala-1	8	Mor	^f^R	C:C	A:G	T:T	G:G	A:G
KS92WGRC20, Ella, AUS28747, KS89WGRC03, KS89WGRC04, Monon, Ribeiro	7	various	R	C:C	A:A	T:T	G:G	A:G
AUS90809, Haala-35	2	USA, Mor	R	**C:T**	A:A	T:T	G:G	A:G
Clark	1	USA	R	C:C	**G:G**	T:T	G:G	A:G
CIGM93.226-0SY	1	Mex	R	C:C	A:A	**C:T**	G:G	A:G
Langdon, CIGM93.203-OSY	2	USA, Mex	R	C:C	A:G	T:T	**C:G**	A:G
KS89WGRC06 (AUS90812)	1	USA	R	C:C	A:A	T:T	**C:C**	**G:G**
CIGM90.906	1	Mex	R	**C:T**	A:G	T:T	**C:G**	A:G
CIGM90.812-03WM-0Y	1	Mex	R	**T:T**	A:G	**C:T**	G:G	A:G
CIGM93.205	1	Mex	R	C:C	**G:G**	T:T	**C:C**	**G:G**
KS93WGRC26 (AUS26929)	1	USA	R	**C:T**	**G:G**	T:T	**C:G**	?
Lola_1, CIGM86.941, CIGM86.941-1B-0B-0B	3	USA, Mex	R	**T:T**	**G:G**	T:T	**C:G**	**G:G**
CIGM90.543, AUS30625	2	Mex	R	**T:T**	**G:G**	**C:T**	G:G	A:G
M6	1	Mex	R	C:C	**G:G**	**C:T**	**C:C**	**G:G**
CIGM86.942-1B-0PR-0, CIGM90.898	2	Mex	R	**T:T**	A:G	**C:C**	**C:C**	**G:G**
AUS26847, CASW02GH00010S, CASS03GH00001S	3	Mex	R	**T:T**	**G:G**	**C:T**	**C:C**	A:G
CIGM86.950, CIGM86.950-1 M-1Y-0B	2	Mex	R	**T:T**	**G:G**	**C:T**	**C:C**	**G:G**

^a^[19]

^b^The SNP1294_*Hfr-2* assay distinguished a ‘C/T’ SNP which is the reverse complement of the ‘G/A’ SNP in the coding sequence

^c^[34]

^d^Refer to Additional file 2: Table S2 for details

^e^*S* = 100% susceptible

^f^*R* = resistant to the Hessian fly biotype in Morocco [19]

Nt in bold indicates genotype associated with the resistant variant/allele of the corresponding gene in the cultivar

This SNP, ‘A/G’ causes an amino acid change from isoleucine to valine in the jacalin-like lectin domain of *Hfr-1* (AAM46813.1).

A short segment of exon I of *Hfr-2* sequenced for the 15 wheat cultivars (see ‘Methods’) gave two SNPs (Table 1, Additional file 1: Table S1) that were observed to occur randomly in the cultivars sequenced. The two primer pairs, Hfr-2_F2a/Hfr-2_R2a and Hfr-2_F2b/Hfr-2_R2b (Table 1), sequenced two different segments, 678 bp and 433 bp, of exon II of *Hfr-2* (Fig. 4).

**Fig. 6:**
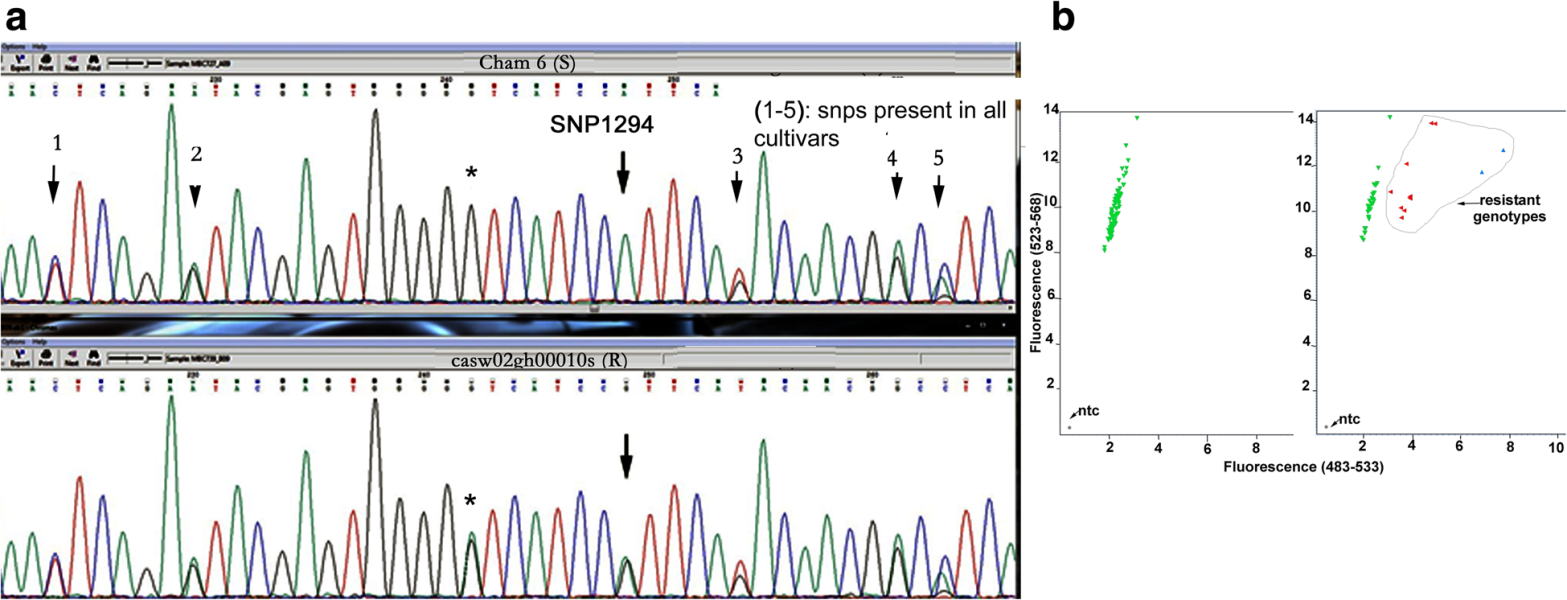
**a** Partial sequence profiles of amplicon from *Hfr-2* with primer pair, Hfr-2_F2b and Hfr-2_R2b, for one R cultivar, CASW02GH00010S, and one S cultivar Cham6. Seventeen SNPs were observed in this segment (Additional file 1: Table S1). Many of these SNPs occur in both cultivars (e.g. SNP labelled ‘1’ to ‘5’) as well as in all the cultivars sequenced (Additional file 1: Table S1). The SNP labelled ‘*’ occurs in the 3rd nucleotide of the codon for glycine and is redundant. The indicated SNP, SNP1294, ‘A/G’, is located at nt position 1294 of the *Hfr-2* mRNA sequence, GenBank AY587018.1 (Fig. 2), and results in an amino acid substitution of isoleucine to valine. This SNP is located in the lectin domain of the gene. A SNP assay, SNP1294_*Hfr-2*, has been developed for high throughput genotyping (Fig. 6b). **b** The endpoint fluorescence in the genotyping of SNP 1294 in exon II of *Hfr-2* using the SNP_1294_*Hfr-2* assay (Table 2) on two different 96-well sample plates. The assay has been designed in the complement orientation and hence the nucleotide called was complementary to the corresponding nt in the gene. The SNP at nt 1294 (AY587018.1) segregates between the S cultivars with ‘T’ (CalFluor, green triangles on y-axis), and the R cultivars with either the ‘C’ (FAM, blue triangles on x-axis) or the heterozygotes (red triangles). This SNP in the coding sequence is ‘G/A’ and results in an amino acid change from isoleucine to valine in the alleles of R cultivars with the gene. No S cultivars have the ‘G’ allele. Twelve of the 39 R cultivars have the ‘G’ allele (Table 3). Refer to section under ‘Results’ for explanation on why the S cultivars are in a tight cluster while the R genotypes are in a loose cluster

Twenty nine SNPs were observed for the first segment of exon II (Additional file 1: Table S1). These SNPs were observed in all the cultivars sequenced, with both nucleotides observed for each SNP in the cultivars sequenced. Seventeen SNPs were observed for the second segment of exon II. Sixteen of these were similarly observed to be present in all the cultivars sequenced (see some examples in Fig. 6a, Additional file 1: Table S1). Only one SNP at nt 1294 was observed to be informative (Table 1, Fig. 6a, b).

The nt 1294 (GenBank AY587018.1) of *Hfr-2* is ‘A’ (Additional file 1: Table S1). The corresponding nucleotide identified in the seven complete alleles from analysis with the whole wheat genome sequence repository (https://wheat-urgi.versailles.inra.fr/Seq-Repository/) is also an ‘A’ nucleotide (Additional file 1: Table S1). Sequencing in this study has found that 2 R cultivars; CIGM90.898 and CASW02GH00010S, possess the heterozygous ‘G:A’ state (Fig. 6a, Additional file 1: Table S1).

A SNP assay, SNP1294_*Hfr-2*, (Table 2) has been designed in this study for high throughput genotyping of the SNP at nt 1294. This assay distinguished a ‘C/T’ SNP which is the reverse complement of the ‘G/A’ SNP in the coding sequence. Two and ten of the 39 R cultivars gave a ‘C:C’ and ‘C:T’ genotype (Fig. 6b, Table 3, Additional file 2: Table S2) respectively, while all the other 237 cultivars have the ‘T:T’ genotype in a tight cluster (Fig. 6b). This supports the findings from whole genome analysis that the nt at this location is an ‘A’ nucleotide for the seven complete alleles found for *Hfr-2* in the wheat genome (Additional file 1: Table S1). The SNP assay (Fig. 6b) showed that the R cultivars in contrast are in a loose cluster, distributed in varying degrees of heterozygosity (Fig. 6b). Whole genome analysis has indicated the gene has 10 alleles (Additional file 1: Table S1) and therefore resistant cultivars can have varying numbers (ranging from 1 to 10) of alleles with the ‘G’ SNP, which explained for the dispersed distribution of the heterozygous R genotypes (Fig. 6b).

Two *H32*-linked markers in Kompetitive Allele Specific PCR (KASP) format (synopGBS901 and IWB65911, [34]) have been genotyped on the set of 249 susceptible and resistant cultivars. These two markers did not give identical results for the cultivars genotyped (Table 3), suggesting that these two markers are not co-located but are in close proximity on the chromosome. The synopGBS901 marker indicated seventeen of the R cultivars have the ‘C:C’ or the heterozygous ‘C:G’ state, suggesting the presence of the *H32* resistance allele, whereas the IWB65911 marker showed only 10 R cultivars gave the ‘G:G’ genotype indicating the presence of the *H32* resistance gene (Table 3). One Australian cultivar, Sunguard is susceptible to Hessian fly but it possesses the heterozygous ‘C:G’ state, indicative of the presence of the *H32* gene (Table 3) with the synopGBS901 marker.

## Discussion

Genetic linkage maps have been used as the framework for the identification of loci and/or genes linked to desirable traits in plant breeding [36, 37]. Mapping populations are thus a very valuable resource for understanding the genetic basis of phenotypic variation and has been applied successfully in wheat for the assignment of quantitative [36] and quality traits [37].

There are many Hessian fly *R* genes and mapping of any new *R* gene will require the establishment of a new population preferably a doubled haploid population. The development of mapping populations for the many *R* genes from different R sources or R cultivars of different origins is laborious, very time-consuming and extremely costly.

This study has thus used an approach to explore genes that have been identified to be specifically induced and expressed in the Hessian fly resistance pathway [16, 17, 27]. The availability of the mRNA sequences of *Hfr-1* and *Hfr-2*, and the whole wheat genome sequence repository (https://wheat-urgi.versailles.inra.fr/Seq-Repository/), facilitated the use of the sequence data for alignment to obtain the structure of the genes (Figs. 1, 2). Primers were designed (Table 1) to re-sequence the target genes for a selected set of wheat cultivars with known phenotypes. This enabled the detection of specific allelic variation in the coding regions of the genes that differentiates selective R wheat sources from S wheat cultivars.

This study has developed two SNP assays, SNP883_*Hfr-1* and SNP1294_*Hfr-2* (Table 2), for the selection of *Hfr-1* and *Hfr-2* respectively. These high throughput SNP genotyping assays have confirmed each SNP identified for some resistant variants of *Hfr-1* and *Hfr-2* by sequencing, on a much larger set of S and R cultivars from different origins (Table 3).

The SNP 883 is located in the jacalin-like lectin domain of *Hfr-1* and SNP 1294 is located in the lectin domain of *Hfr-2*. Plant lectins and proteins with one or more lectin domains represent a major part of plant receptors in plant defense system [38].

The two SNP assays, SNP883_*Hfr1* and SNP1294_*Hfr-2,* differentiated 14 and 12 R cultivars from the 210 S wheat cultivars (Table 3) respectively. The SNP1294_*Hfr-2* assay distinguished a ‘C/T’ SNP (Table 2) which is the reverse complement of the ‘G/A’ SNP in the coding sequence. Thus both SNPs are ‘G/A’ and resulted in an amino acid change from isoleucine (ATC or ATT) to valine (GTC, GTT) in the lectin domain of the proteins of gene alleles of the resistant cultivars. None of the S cultivars possesses the nucleotide that codes for valine in these two alleles (Table 3). Both are branched chain amino acids and a substitution in the lectin domain is postulated to cause a change in the molecular form of the protein and sensitivity of the protein receptor to elicitor molecules. A single base change in a conserved domain of a target gene has been reported to change host resistance to elicitors [30–33], and changes to the plant phenotype [39]. Changes in the protein structure will result in changes in elicitor recognition and signal activation in the resistance pathway of the plant defence system.

Six R cultivars have been found to have the ‘G’ SNP only in the *Hfr-1* allele and another four different R cultivars have the ‘G’ SNP only in the *Hfr-2* allele (Table 3). Another eight different R cultivars of the 39 cultivars have the ‘G’ SNP in both gene alleles (Table 3). Thus, altogether eighteen of the 39 resistant cultivars genotyped have either or both of the *Hfr-1* and *Hfr-2* alleles associated with R cultivars only. Twenty one R cultivars do not have either of the ‘G’ SNP in both *Hfr-1* and *Hfr-2* alleles. These cultivars thus represent different sources of R germplasm.

The *R* gene, *H32*, confers resistance to the highly pervasive Biotype L of Hessian fly and was mapped on chromosome 3DL [7]. This study has found that the two *H32-*linked SNP markers [34] gave different results for the same R cultivars (Table 3) suggesting that these markers are not co-located on the chromosome. This has also been illustrated in the Australian cultivar, Sunguard, which gave different results for the two markers. The synopGBS901 marker indicated the presence of the *H32* gene whereas the IWB65811 marker indicated an absence (Table 3). It was shown by screening with a Hessian fly population from the Settat region in Morocco [19] to be susceptible. The Hessian fly has many biotypes and had been reported to give different responses to different *R* genes [40]. It is thus uncertain whether the *H32* gene is absent in Sunguard (indicated by the IWB65811 marker) or the single gene (indicated by the synopGBS901 marker) in Sunguard was not effective against the biotype from Morocco. It would thus be of interest to screen Sunguard with other biotypes of Hessian fly in the USA for its resistance response.

The genotype profiles of the thirty nine R cultivars (Table 3) indicated some cultivars with single *R* allele of any of the three Hessian fly response genes; *HfrDrd*, *Hfr-1* or *Hfr-2,* or the *H32* gene. The Hessian fly response genes are multi-allelic and are located on different wheat chromosomes (Table 3). Resistant cultivars with 1, 3 or 4 *R* genes in various combinations have also been identified (Table 3).

Work is being pursued to develop doubled haploid populations from crosses between premium S wheat cultivars and suitable R cultivars identified from outcomes in this study, for both the re-confirmation of the SNP markers that have been developed and utilization of R lines from the populations for breeding. In addition, there is a great potential for the application of gene editing technology using the CRISPR/Cas9 system [41] to achieve the desired mutations to produce resistant alleles of *Hfr-1* and *Hfr-2*.

## Conclusions

This study has targeted two Hessian fly response genes, *Hfr-1* [17] and *Hfr-2* [27], which have been reported to be induced specifically in response to a Hessian fly attack, to identify allelic differences between S and R cultivars. Resequencing exons of *Hfr-1* and *Hfr-2,* have identified a SNP in the lectin domain of each gene that segregates some R sources from S cultivars. Each of the two SNPs identified in *Hfr-1* and *Hfr-2* are ‘G/A’ and resulted in an amino acid change from isoleucine to valine in the lectin domain of the proteins of the alleles in the R cultivars. Two SNP assays have been developed for the selection of *Hfr-1* and/or *Hfr-2,* and with the published assays for *HfrDrd* and the *H32* gene, can be used for the selection and incorporation of one or more of these 4 *R* genes identified in the different R sources in wheat breeding programs.

## Methods

### Wheat materials

Wheat materials in this study were obtained from the Australian Winter Cereals Collection (AWCC), Tamworth, Australia (now The Australian Grains Genebank, Horsham). They included 249 wheat cultivars identified in previous studies [42, 43]. The extraction of DNA from wheat leaves were as described previously [19].

### Screening for hessian fly resistance

A Hessian fly population from the Settat region in Morocco was used to screen for resistance according to the published protocol [42]. The cultivars, Arrehane and Rajae, were used as the R and S checks respectively.

### Amplification and sequencing of gene exons in *Hfr-1* and *Hfr-2*

The complete mRNA sequences of *Hfr-1* (AF483596.1) and *Hfr-2* (AY587018.1) were analysed by BLAST against the wheat whole genome sequence repository (https://wheat-urgi.versailles.inra.fr/Seq-Repository/) to retrieve homologous and homeologous genomic sequences. This has enabled the alignment of the mRNA and the genomic sequences to determine the location of the exons and introns in the gene (Figs. 1 and 2) to allow the design of primers for the amplification and sequencing of the exons (Table 1).

Exon segments from both genes were amplified with appropriate primer pairs (Table 1) from 7 S wheat accessions (AGT-Young, Axe, Cham-6, Carnamah, Newton, Janz, Frame) and eight different R accessions (Clark, Ella, CIGM90.898, CIGM90.906, Haala-35, AUS26929, Monon and CASW02GH00010S). Amplification and sequencing were performed as described previously [19], with the exception that the extension phase of the PCR cycle for the four exon segments of *Hfr-1* and the exon segment from Hfr-2_F2a/R2a was 35 cycles of 94 °C for 20 s (denaturation), 73 °C for 30 s with the annealing temperature decreasing by 1 °C /cycle to 65 °C.

The SNPs observed in the sequence alignment of the exon segments sequenced for the various cultivars were documented with reference to the nt positions of the reference mRNA sequences of *Hfr-1* (AF483596.1, Additional file 1: Table S1) and *Hfr-2* (AY 587018.1, Additional file 1: Table S1).

### SNP genotyping

Two SNP genotyping assays have been developed, one each for *Hfr-1* and *Hfr-2* (Table 2). The SNP assay for exon IV of *Hfr-1* was designed on the Taqman format with Life Technologies (https://www.thermofisher.com) and the SNP assay for exon II of *Hfr-2* was designed with the SNP design software at Biosearch Technologies (https://www.biosearchtech.com). The assays were performed according to supplier’s instructions on the Roche LightCycler480 real-time PCR machine.

Three additional SNP assays, two *H32*-linked markers in KASP format (synopGBS901 and IWB65911, [34]) and a SNP assay for *HfrDrd* [19] were also performed on the same set of 249 wheat cultivars.

## Additional files

Additional file 1: **Table S1.** List of SNPs documented with reference to mRNA sequences of *Hfr-1* (AF483596.1) and *Hfr-2* (AY587018). (XLSX 32 kb)

Additional file 2: **Table S2.** Genotype data of Hessian fly response genes, *HfrDrd*, *Hfr-1* and *Hfr-2* and the resistance gene *H32,* for 210 S and 39 R wheat cultivars. (XLSX 24 kb)

## Abbreviations

Aus: Australia
BLAST: Basic local alignment search tool
*Hfr-1*: Hessian fly response gene 1
*Hfr-2*: Hessian fly response gene 2
KASP: Kompetitive Allele Specific PCR
Mex: Mexico
Mor: Morocco
mRNA: Messenger RNA
nt: Nucleotide
*R*: Resistance gene
R: Resistant
S: Susceptible
SNP: Single nucleotide polymorphism
